# Analytical Performance Characteristics of a New Transcription-Mediated Amplification Assay for Treponema pallidum

**DOI:** 10.1128/JCM.00511-21

**Published:** 2021-07-19

**Authors:** Damon Getman, Mike Lin, Nesreen Barakat, Rhonda Skvoretz, Charmie Godornes, Paul Swenson, Ashley Nenninger, Matthew R. Golden, Sheila A. Lukehart

**Affiliations:** a Hologic, Inc., San Diego, California, USA; b Public Health—Seattle and King County, Seattle, Washington, USA; c Division of Allergy and Infectious Diseases, Department of Medicine, University of Washington School of Medicine, Seattle, Washington, USA; Marquette University

**Keywords:** syphilis, *Treponema pallidum*, Aptima, TMA, NAAT

## Abstract

This study evaluated the performance characteristics of a new research-use-only transcription-mediated amplification (TMA) assay for the detection of rRNA from Treponema pallidum. Analytical sensitivity determined using dark-field microscopy-quantitated T. pallidum was 1.4 organisms/ml (95% confidence interval [CI], 0.7 to 6.33 organisms/ml). Dilution of *in vitro*-transcribed (IVT) T. pallidum RNA in Aptima sample transport medium (STM) yielded 100% positivity (*n* = 3) at 10 copies/ml (4 copies/reaction). Analytical specificity testing of nontarget microorganisms (*n* = 59), including the closely related nonsyphilis treponemes Treponema denticola and Treponema phagedenis, yielded 0% positivity. TMA testing of mucosal swab specimens collected from men who have sex with men (MSM) attending a sexually transmitted disease clinic yielded 1.8% (17/944) positive results. A collection of 56 serum specimens obtained from a separate cohort of patients with known rapid plasma reagin (RPR) statuses and clinical diagnoses of syphilis was 19.6% (11/56) TMA positive overall and 29.7% (11/37) positive among subjects with syphilis diagnoses, including 8 (36.3%) of 22 persons with primary or secondary syphilis, 2 (20%) of 10 persons with early latent syphilis, and 1 (20%) of 5 persons with late latent or unstaged syphilis. None (0%) of the 18 RPR-positive sera from patients with histories of treated syphilis were TMA positive. These results show that TMA is an analytically sensitive and specific method for the detection of T. pallidum rRNA and is compatible with serum specimens in addition to pharyngeal and rectal mucocutaneous swab specimens. Automated real-time TMA testing for T. pallidum may be useful as an adjunctive method with serology for screening and diagnostic testing of selected patient populations for syphilis.

## INTRODUCTION

Syphilis is making an unfortunate comeback. Rates of infection have increased steadily over the past 20 years (1), with the majority of cases occurring in men who have sex with men (MSM). However, in recent years, rates have increased substantially in women and in men who have sex with women (MSW); congenital syphilis has risen dramatically; and case reporting from non–sexually transmitted disease (non-STD) clinics has also increased (1), indicating that this epidemic is moving beyond MSM into additional vulnerable populations. The United States is not alone in this trend; syphilis infection is on the rise worldwide (2–4). With the coronavirus disease of 2019 (COVID-19) pandemic disrupting diagnostic supply chains, limiting in-person health care visits, and redirecting public health resources away from sexually transmitted infection (STI) prevention and care, STI screening has been significantly constrained over the past year, which has the potential to exacerbate these already concerning upward trends.

Syphilis is caused by the bacterium Treponema pallidum subsp. *pallidum*, is spread via sexual contact with an infected person, and leads to a multistage disease. Due to the organism’s slow growth, there is an extended incubation period before the development of a primary lesion, which can range from 10 days to 3 months (1, 5). Lesions are frequently painless and may develop in locations where they are undetected, creating opportunities for further transmission during the primary stage of infection (5, 6). The most common indirect method used for syphilis diagnosis is serological testing, but the time between infection and seroconversion creates a diagnostic window period during which screening tests may yield false-negative results, facilitating the spread of infection from persons with subclinical infections. Like primary and secondary syphilis, rates of early latent and unknown-duration/late-stage syphilis have also been on the rise in recent years, indicating that primary syphilis and secondary syphilis often go undetected or are misdiagnosed and left untreated to progress to later-stage disease (1, 7).

A sensitive and specific molecular test could be a valuable supplement to serology in the ability to diagnose syphilis, particularly during the early stages, by filling the serological diagnostic gap between transmission and seroconversion. A number of syphilis PCR assays have been developed, and studies have shown that as many as 27% of primary syphilis cases could be identified by PCR prior to seroconversion (8–12). Recently, we used an investigational transcription-mediated amplification (TMA) assay in a research study evaluating direct detection of T. pallidum rRNA by TMA in extragenital mucosal swabs, showing that TMA can sometimes detect syphilis in patients with negative serological results (13). Here, we present data on the analytical performance of this investigational real-time TMA assay, including analytical sensitivity, specificity, and interference. Rectal swabs, pharyngeal swabs, and serum as clinical sample types for the detection of T. pallidum by TMA were also investigated.

## MATERIALS AND METHODS

### Real-time TMA assay design.

A region of domain 1 of T. pallidum 23S rRNA was utilized for the assay target region to maximize RNA sequence differences between T. pallidum and other, nonsyphilis commensal treponeme species. Target capture, primer, and fluorescent probe (6-carboxyfluorescein [FAM]-labeled molecular torch) oligonucleotides (spike volume, 50 μl each) were combined with Aptima-format real-time TMA general-purpose reagents prior to the loading of the reagents on the Panther system instrument (both from Hologic, Inc., San Diego, CA). All TMA assay testing was performed on the Panther system using a specimen volume of 0.4 ml per reaction and a predefined T-time (fluorescence signal emergence time, in minutes) cutoff value of 40 min and 1,000 relative fluorescent units (RFU). The assay used in this study was for research use only (RUO), not for use in diagnostic procedures.

### Analytical performance studies.

Panels of microorganisms for analytical sensitivity and specificity validation studies were diluted in Aptima sample transport medium (STM) at various concentrations prior to testing. For analytical sensitivity (limit of detection [LOD]) determination, dark-field microscopy-quantitated T. pallidum subsp. *pallidum* (Nichols strain) organisms were diluted serially in STM to create seven positive panels ranging from 3 to 0.003 organisms/ml and were tested with an STM blank panel using 10 replicates each for all panels. Positivity results were analyzed by probit analysis (normal model) to determine the 50% and 95% LOD values. Also tested were replicates (*n*, 3 each) of panels consisting of an *in vitro*-transcribed (IVT) RNA target encompassing a sequence corresponding to domain 1 of T. pallidum 23S rRNA, diluted in STM so as to contain 300 to 10 copies/ml. Analytical sensitivity was confirmed by testing five replicates of dark-field microscopy-quantitated T. pallidum subsp. *pallidum* diluted to 1 organism/ml in a matrix consisting of pooled anorectal swabs in STM. These swabs were collected from consenting healthy donors at Hologic, and one swab from each donor was added to 3 ml of Aptima STM. For analytical specificity (cross-reactivity and inhibition) studies, 59 nontarget bacterial, fungal, and viral species, including nonsyphilis treponeme species closely related to T. pallidum, were added to STM at the highest concentration feasible (1 × 10^5^ to 1 × 10^6^ CFU/ml, 50% tissue culture infective doses [TCID_50_]/ml, or copies/ml) in the absence or presence of a low titer (30 copies/ml) of IVT T. pallidum 23S rRNA and were tested with the assay. For additional assessment of the cross-reactivity potential of the assay with nonsyphilis treponeme species not available for assay testing, *in silico* analysis was performed by Clustal W alignment of 23S rRNA sequences from 15 Treponema species to determine the percentage of base difference in the corresponding TMA 23S rRNA target region of each organism from the T. pallidum 23S target region reference sequence.

### Clinical specimen testing.

As reported previously (13), deidentified residual rectal and pharyngeal specimens were obtained and prepared from 534 men who have sex with men (MSM) attending the Public Health—Seattle & King County (PHSKC) Sexual Health Clinic (SHC) from September to November 2017. The study was reviewed and approved by the University of Washington IRB, with an exemption from consent for testing remnant deidentified specimens. Briefly, consecutive clinician-collected and patient-collected Aptima Multitest swab specimen tubes previously tested for gonorrhea and chlamydia using the Aptima Combo 2 assay had patient identifying information removed by laboratory staff and were subsequently relabeled with a study identification number and stored at –20°C prior to direct testing with the T. pallidum TMA assay. TMA-positive swab specimens, as well as swab specimens from 20 randomly selected patients with negative TMA test results, were also tested using a T. pallidum PCR assay for the single-copy-number T47 gene (*TP0574*) (14). The results of this PCR testing were reported previously (13) in a companion study to the current investigation but are included in the present analysis in a revised format to provide for ready comparison within the context of the new analytical testing results. A convenience set of 56 remnant serum specimens with known rapid plasma reagin (RPR) results obtained from a separate cohort of clinic patients was tested by adding 0.5 ml of serum to a specimen transport tube containing 0.5 ml of Aptima urine transport medium (UTM), followed by the addition of 1.0 ml of Aptima STM prior to analysis on the Panther instrument. All T. pallidum TMA assay testing was performed at Hologic by operators who were blinded to all patient- and specimen-related information, including STD status and syphilis serology results.

## RESULTS

A research use transcription-mediated amplification (TMA) assay was developed to detect T. pallidum rRNA using Aptima-format reagents on the automated Panther system. The assay utilizes sequence-specific magnetic-bead-based target capture and isothermal real-time TMA to capture, amplify, and detect a region of domain 1 of T. pallidum 23S rRNA.

The analytical sensitivity of the TMA assay was determined by testing replicates of contrived panels of either dark-field microscopy-quantitated T. pallidum whole organisms or IVT RNA encompassing domain 1 of T. pallidum 23S rRNA, each diluted serially in STM. The results (Table 1) show that TMA was able to detect 1 T. pallidum organism/ml at 90% reactivity and 3 T. pallidum organisms/ml at 100% (*n*, 10 each). Among the synthetic IVT RNA target panels, TMA reactivity was 100% for all panels with concentrations ranging from 300 to 10 copies/ml (*n*, 10 each). TMA assay sensitivity was confirmed by achieving 100% reactivity (*n* = 5) for T. pallidum organisms spiked to a concentration of 1 organism/ml into a pooled-anorectal-swab clinical-specimen matrix. The 50% and 95% limits of detection obtained by probit analysis of the reactivity data were 0.273 T. pallidum organism/ml (95% confidence interval [CI], 0.157 to 0.475) and 1.36 T. pallidum organisms/ml (95% CI, 0.705 to 6.328), respectively (Table 1).

**TABLE 1:** Analytical sensitivity of the Treponema pallidum TMA assay for the detection of T. pallidum RNA targets^*a*^

Panel (unit of measurement)	Specimen matrix	Concn	No. of specimens	No. (%) positive^*b*^
T. pallidum (organisms/ml)	STM	0.003	10	0 (0)
		0.01	10	0 (0)
		0.03	10	0 (0)
		0.1	10	2 (20)
		0.3	10	5 (50)
		1	10	9 (90)
		3	10	10 (100)
T. pallidum IVT 23S rRNA target (copies/ml)	STM	10	3	3 (100)
		30	3	3 (100)
		100	3	3 (100)
		300	3	3 (100)
T. pallidum (organisms/ml)	Pooled anorectal swabs in STM	1	5	5 (100)

a STM, Aptima specimen transport medium; IVT, *in vitro* transcribed.

b By probit analysis, the LOD_50_ is 0.273 (95% CI, 0.157 to 0.475) organism/ml and the LOD_95_ is 1.36 (95% CI, 0.705 to 6.328) organisms/ml.

Analytical specificity (cross-reactivity and interference) for the TMA assay was assessed by testing 59 nontarget bacterial, fungal, and viral species at high titers in the absence or presence of low concentrations of either T. pallidum organisms or IVT 23S rRNA (Table 2). None of the nontarget organisms interfered with detection of the low-titer (30 IVT rRNA copies/ml or 30 T. pallidum organisms/ml) T. pallidum RNA targets. All panels lacking the spiked T. pallidum RNA target yielded 0% positive TMA results except for those containing T. pallidum subsp. *pertenue*, T. pallidum subsp. *endemicum*, and T. paraluiscuniculi, all of which were 100% positive by TMA, as expected due to 100% sequence identity of the 23S rRNA from those species with the 23S rRNA from T. pallidum subsp. *pallidum*. Two human nonsyphilis treponeme species, Treponema phagedenis and Treponema denticola, which exhibit 18% and 27% RNA base sequence difference, respectively, with T. pallidum 23S rRNA in the TMA target region, were nonreactive (0% positive) by TMA. *In silico* analysis of published 23S rRNA sequences from an additional 10 nonsyphilis treponeme species not available for testing showed 27% to 59% RNA base sequence differences from the TMA T. pallidum 23S rRNA target region, suggesting a low risk of cross-reactivity of the TMA assay with these nontarget organisms (see Table S1 in the supplemental material).

**TABLE 2:** Cross-reactivity and interference/inhibition of the Treponema pallidum TMA assay for nontarget organisms

				% positive:	
Specificity panel	Organism	Final concn	Unit of measurement	Without T. pallidum IVT RNA	With T. pallidum IVT RNA^*a*^ (30 copies/ml)
1	Acinetobacter lwoffii (ATCC 15309)	1.00E+06	CFU/ml	0	100
	Actinomyces israelii (ATCC 12102)	5.00E+09	rRNA copies/ml		
	Alcaligenes faecalis (ATCC 8750)	1.00E+06	CFU/ml		
	Atopobium vaginae (ATCC BAA-55)	5.00E+09	rRNA copies/ml		
2	Bacteroides fragilis (ATCC 25285)	1.00E+06	CFU/ml	0	100
	Bifidobacterium adolescentis (ATCC 15703)	1.00E+06	CFU/ml		
	Campylobacter jejuni (ATCC 33560)	1.00E+06	CFU/ml		
	Chlamydia trachomatis (ATCC VR-878)	1.00E+05	IFU/ml		
3	Candida krusei*(*ATCC 14243)	1.00E+06	CFU/ml	0	100
	Candida lusitaniae*(*ATCC 34449)	1.00E+06	CFU/ml		
	Clostridium difficile (ATCC 9689)	1.00E+06	CFU/ml		
	Corynebacterium genitalium (ATCC 33030)	1.00E+06	CFU/ml		
4	Cryptococcus neoformans (ATCC 32045)	1.00E+06	CFU/ml	0	100
	Eggerthella lenta (ATCC 25559)	1.00E+06	CFU/ml		
	Enterobacter cloacae (ATCC 13047)	1.00E+06	CFU/ml		
	Enterococcus faecalis (ATCC 19433)	1.00E+06	CFU/ml		
5	Escherichia coli (ATCC 11775)	1.00E+06	CFU/ml	0	100
	Haemophilus ducreyi (ATCC 33940)	1.00E+06	CFU/ml		
	Klebsiella pneumoniae (ATCC 23357)	1.00E+06	CFU/ml		
	Listeria monocytogenes (ATCC 15313)	1.00E+06	CFU/ml		
6	Lactobacillus acidophilus (ATCC 4356)	1.00E+06	CFU/ml	0	100
	Lactobacillus iners (ATCC 55195)	1.00E+06	CFU/ml		
	Lactobacillus mucosae (ATCC 43179)	1.00E+06	CFU/ml		
	Leptotrichia buccalis (ATCC 14201)	1.00E+06	CFU/ml		
7	Mobiluncus curtisii*(*ATCC 35241)	5.00E+09	rRNA copies/ml	0	100
	Mycoplasma genitalium (ATCC 49895)	1.00E+06	CFU/ml		
	Mycoplasma hominis (ATCC 23114)	5.00E+09	rRNA copies/ml		
	Neisseria gonorrhoeae (ATCC 19424)	1.00E+06	CFU/ml		
8	Peptostreptococcus magnus (ATCC 29328)	1.00E+06	CFU/ml	0	100
	Prevotella bivia (ATCC 29303)	1.00E+06	CFU/ml		
	Propionibacterium acnes (ATCC 6919)	1.00E+06	CFU/ml		
	Proteus vulgaris (ATCC 8427)	1.00E+06	CFU/ml		
9	Staphylococcus aureus (ATCC 12600)	1.00E+06	CFU/ml	0	100
	Staphylococcus epidermidis (ATCC 14990)	1.00E+06	CFU/ml		
	Streptococcus agalactiae*(*ATCC 13813)	1.00E+06	CFU/ml		
	Streptococcus pyogenes*(*ATCC 12344)	1.00E+06	CFU/ml		
10	Trichomonas vaginalis (ATCC 30236)	1.00E+05	Cells/ml	0	100
	Ureaplasma parvum (ATCC 27813)	1.00E+06	CFU/ml		
	Ureaplasma urealyticum (ATCC 27618)	1.00E+06	CFU/ml		
11	Herpes simplex virus I (ATCC VR-260)	1.00E+05	TCID_50_/ml	0	100
	Herpes simplex virus II (Zeptometrix 0810220CF)	1.00E+05	TCID_50_/ml		
	HIV-1 (Hologic BI0065)	1.00E+06	rRNA copies/ml		
12	Candida dubliniensis (ATCC MYA-646)	1.00E+06	CFU/ml	0	100
	Candida albicans (ATCC 90028)	1.00E+06	CFU/ml		
	Candida glabrata (ATCC 32554)	1.00E+06	CFU/ml		
	Candida parapsilosis (ATCC 99018)	1.00E+06	CFU/ml		
	Candida tropicalis (ATCC 34139)	1.00E+06	CFU/ml		
13	Gardnerella vaginalis (ATCC 14018)	1.00E+06	CFU/ml	0	100
	Lactobacillus crispatus (ATCC 33820)	1.00E+06	CFU/ml		
	Lactobacillus gasseri (ATCC 33323)	1.00E+06	CFU/ml		
	Lactobacillus jensenii (ATCC 25258)	1.00E+06	CFU/ml		
14	Treponema denticola (ATCC 33520)	1.00E+06	Cells/ml	0	100^*b*^
15	Treponema phagedenis biotype Reiter	1.00E+06	Cells/ml	0	ND^*c*^
16	Treponema pallidum subsp. *pertenue* Gauthier	<1.60E+08	rRNA copies/ml	100	ND
17	Treponema pallidum subsp. *pertenue* Samoa D	<1.60E+08	rRNA copies/ml	100	ND
18	Treponema pallidum subsp. *pertenue* Samoa F	<1.60E+08	rRNA copies/ml	100	ND
19	Treponema pallidum subsp. *endemicum* Bosnia A	<1.60E+08	rRNA copies/ml	100	ND
20	Treponema pallidum subsp. *endemicum* Iraq B	<1.60E+08	rRNA copies/ml	100	ND
21	Treponema paraluiscuniculi Cuniculi A (C16419)	1.00E+06	rRNA copies/ml	100	ND

a Copies of *in vitro*-transcribed RNA corresponding to T. pallidum 23S rRNA.

b T. pallidum organisms at 30 organisms/ml.

c ND, not done.

**FIG 1:**
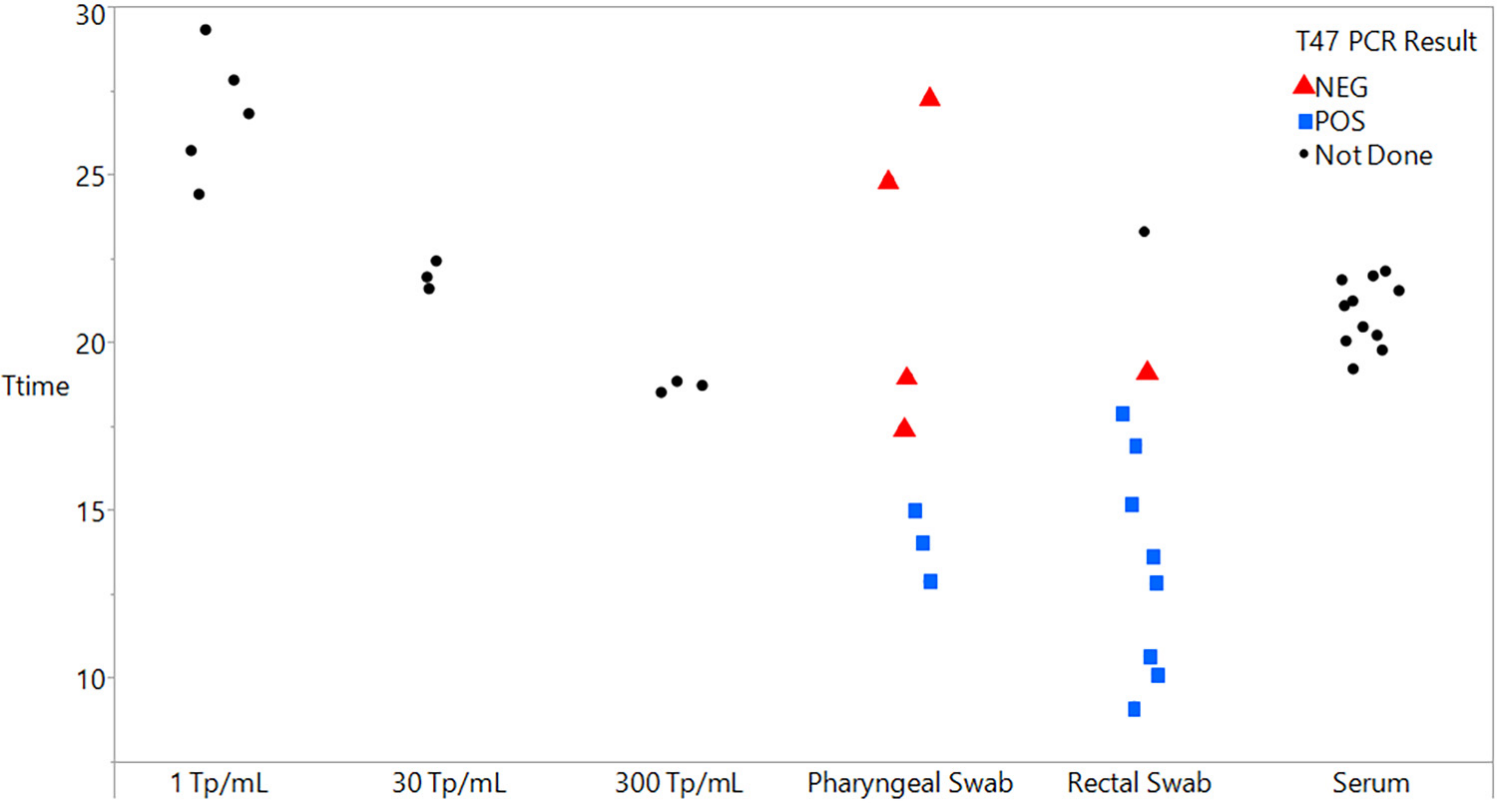
Distribution of Treponema pallidum TMA assay T-time values (in minutes) with corresponding T47 PCR results from testing of pharyngeal swab, rectal swab, and serum clinical specimens. Control panels contain dark-field microscopy-quantitated T. pallidum organisms.

To evaluate the performance of TMA at detecting T. pallidum in clinical specimens, remnant oropharyngeal swabs, anorectal swabs, and serum specimens obtained from MSM attending an STD clinic were tested with the assay on the Panther system instrument. The T-time values (expressed in minutes) of TMA-positive results for serum and swab specimens, as well as for dark-field microscopy-quantitated T. pallidum control panels, are shown in Fig. 1. Mean (range) T-times for oropharyngeal swab, anorectal swab, and serum specimens were 18.63 (14.03 to 27.24), 14.84 (9.14 to 23.25), and 20.85 (19.2 to 22.11) min, respectively. Mean and range T-time values for T. pallidum organism control panels diluted in STM to 1, 30, and 300 T. pallidum organisms/ml were 26.8 (24.4 to 29.3), 22.0 (21.6 to 22.4), and 18.7 (18.5 to 18.8) min, respectively.

For the rectal and pharyngeal swab specimens, correlation of TMA-positive and -negative results with PCR, RPR serology, and clinical diagnosis of syphilis has been reported previously (13). Table 3 shows the agreement between TMA and T47 PCR assays for 75 of these swab specimens. The overall agreement between TMA and T47 PCR for the swab specimens was 92% (69/75), with positive and negative agreements of 100% (11/11) and 92.1% (58/63), respectively.

**TABLE 3:** Agreement of TMA and T47 PCR nucleic acid amplification tests for T. pallidum detection in mucosal swab specimens^*a*^

TMA	No. of specimens with the indicated result by T47 PCR									
	Rectal swab			Pharyngeal swab			Combined swabs			
	Positive	Negative	Not done	Positive	Negative	Not done	Positive	Negative	Not done	Total
Positive	8	1^*b*^	1	3	4^*c*^	0	11	5	1	17
Negative	0	27	0	0	31	0	0	58	0	58
Not done	0	0	2	0	0	0	0	0	2	2
Total	8	28	3	3	35	0	11	63	3	77

a Positive percent agreement, 100% (11/11); negative percent agreement, 92.1% (58/63); overall percent agreement, 92% (69/75).

b Includes one person with early latent syphilis.

c Includes one person with primary, one with secondary, and one with late latent syphilis, as well as one person without a syphilis diagnosis.

The correlation of the results of TMA testing of the RPR-positive serum specimens with clinical diagnoses is presented in Table 4. TMA positivity was 29.7% (11/37) among persons with syphilis diagnoses. TMA was 37.5% (3/8) positive for persons with primary syphilis, 35.7% (5/14) positive for secondary syphilis, and 20% (2/10) positive for persons diagnosed with early latent syphilis. There was one TMA-positive result for a serum specimen from a person with a clinical diagnosis of syphilis with no staging indicated in the medical record. TMA was 0% (0/19) positive with sera from persons with histories of treated syphilis (*n* = 18) or with a biological false-positive RPR result (*n* = 1).

**TABLE 4:** Serum specimen testing by the Treponema pallidum TMA assay

TMA result	No. with the following syphilis diagnosis:							
	History of syphilis^*a*^	Primary	Secondary	Early latent	Late latent	Syphilis, unspecified^*b*^	Biological false positive	Total
Positive	0	3	5	2	0	1	0	11
Negative	18	5	9	8	4	0	1	45
Total no. (% positive)	18 (0)	8 (37.5)	14 (35.7)	10 (20)	4 (0)	1 (100)	1 (0)	56 (19.6)

a Seropositive sera from treated patients.

b Stage not specified in the medical record.

## DISCUSSION

Syphilis laboratory diagnosis has traditionally relied on serology-based methods for indirect detection of treponeme infection, with dark-field microscopy playing a minor role as a direct detection method at the point of care in rare clinical settings with that capability. However, compared to the high (>95%) sensitivity of serology in secondary and latent syphilis, the sensitivity of serology during primary disease is diminished, with studies showing negative serology results among infected persons ranging from 14% to 29%, depending on the method used (15–17). With the increased incidence of syphilis cases in various populations worldwide, the addition of new direct diagnostic methods, such as nucleic acid amplification tests (NAATs), to testing algorithms has been proposed to improve syphilis case-finding, especially for the detection of early primary syphilis (18–20).

This study evaluated the performance characteristics of a new research use real-time transcription-mediated amplification assay for the detection of 23S rRNA of T. pallidum. The results show that TMA is an analytically sensitive and specific method for T. pallidum detection, with a 95% limit of detection of approximately 0.5 treponeme/reaction and 4 copies of IVT 23S rRNA/reaction. The T. pallidum TMA assay also does not cross-react with a wide range of nontarget viral, fungal, and bacterial species, including closely related nonsyphilis human treponeme species T. phagedenis and T. denticola. However, as expected, the assay cannot distinguish T. pallidum subsp*. pallidum* from T. pallidum subsp*. pertenue*, T. pallidum subsp*. endemicum*, and the rabbit pathogen T. paraluiscuniculi, because these organisms, though rarely encountered in routine laboratory testing, all share 100% sequence identity in the 23S rRNA target region of the assay.

Clinical specimen testing showed that the assay is compatible with oropharyngeal swab, anorectal swab, and serum sample matrices, with T-time values ranging from approximately 9 to 27 min for these three specimen types, and a high overall agreement (92%) of T47 PCR with TMA for the swab specimens. While the assay is not validated to be quantitative, tight clustering of the serum specimen T-times around a mean value of 20.8 min indicates the presence of treponemes possibly at lower concentrations in these serum samples than in some of the swab-based specimens, a finding consistent with previously published reports (21–22). Our finding of a relatively low positive agreement (20% to 37%, depending on clinical stage) between RPR serological status and serum TMA results in this convenience sample of serum specimens is consistent with previous data using PCR to test serum, for which sensitivity estimates ranged from 14.7% to 50.2% (8, 22, 23).

The results presented in this report build on our prior work (13) and demonstrate the high analytical sensitivity and specificity of TMA for the detection of T. pallidum, especially the absence of cross-reactivity of the assay with nonsyphilis treponemes T. denticola and T. phagedenis and the ability to detect T. pallidum RNA in serum. The use of PCR-based NAATs as a direct detection method for syphilis has been reported on extensively, with several studies showing that these assays can detect T. pallidum in seronegative persons (8–12, 24, 25). In our companion study to this current investigation, TMA also detected an additional 10% of syphilis infections in seronegative persons, a finding confirmed by T47 PCR testing of the same samples (13). Combined, these results suggest that molecular testing may be a useful adjunctive method to serological screening by enabling the detection of occult infection in the preseroconversion window period prior to the onset of symptomatic primary syphilis, as well as for persons with recently developed chancres. The asymptomatic period can last from 10 to 90 days, during which time the mucosal shedding of T. pallidum could theoretically lead to partner transmission and infection. Adding NAAT testing of mucosal swab-based samples to current serology-based screening algorithms may therefore help close the “window period gap” from initial infection to seroconversion. Although empirical evidence for this approach is currently lacking, clinicians and public health programs have sometimes employed a similar strategy for HIV screening using pooled NAAT testing (26). Such a strategy may be particularly useful in geographic areas or patient populations with high incidences of syphilis (18).

This study is limited by the lack of confirmatory testing by a second or third sensitive NAAT of the small number of swab and serum samples evaluated. We were also unable to test more than a few nonsyphilis treponeme species for cross-reactivity by TMA and instead have relied on an *in silico* base difference analysis of 23S rRNA sequences for making projections as to the clinical specificity of the assay. However, the nonsyphilis treponeme species that tested negative by TMA have the highest sequence identity of the target region 23S rRNA with T. pallidum 23S rRNA among those species with described genomes, providing assurance as to the fidelity of the assay for detecting T. pallidum species.

In conclusion, we have developed a fully automated investigational real-time TMA assay that is sensitive and specific for the detection of T. pallidum rRNA. This technology may be useful for research studies aimed at defining optimal clinical sampling methods and screening algorithms for selected populations at high risk of contracting syphilis.
