# Supplementary material for: Analytical Performance Characteristics of a New Transcription-Mediated Amplification Assay for Treponema pallidum
Source: J Clin Microbiol. 2021 Jul 19;59(8):e00511-21. doi: 10.1128/JCM.00511-21 (PMC8373238; doi:10.1128/JCM.00511-21)
Supplement: Supplemental file 1 — Table S1. Download JCM.00511-21-s0001.pdf, PDF file, 34 KB [file jcm.00511-21-s0001.pdf]

Table S1. *In silico* nucleotide base difference analysis of 23S rRNA sequences from various treponeme species compared to *T. pallidum* ssp. *pallidum* in the 23S rRNA target region of the *Treponema pallidum* TMA assay.

**Species (Strain; NCBI Genbank Accession Number)**	**% base difference from *T. pallidum* ssp. *pallidum* in TMA assay target region**	**TMA % cross reactivity (n=3)** [a]
T. pallidum ssp. *endemicum* (Samoa F; CP007548.1)	0	100
T. pallidum ssp. *pertenue* (CDC2; CP002375.1)	0	100
T. paraluiscuniculi (Cuniculi A; JX120547.1)	0	100
T. phagedenis (4A; AQCFO1)	18	0
T. denticola (OTK; AGDYO1)	27	0
T. succinifaciens (DSM 2489; CP002631.1)	27	NT
T. primitia (ZAS-2; NR_076709.1)	28	NT
T. pedis (T M1; AOTMO1)	28	NT
T. caldarium (H1; NR_076899.1)	30	NT
T. azotonutricium (ZAS-9; NR_076708.1)	30	NT
T. socranskii (ssp. *paredis*; NZ_KE332516.1	34	NT
T. putidum (OMZ758; CP009228.1)	38	NT
T. brennaborense (DD5/3; NR_076878.1)	40	NT
T. lecithinolyticum (ATCC700322; NZ_KI260563.1)	41	NT
T. maltophilum (ATCC 51939; ATFF01000006.1)	59	NT

[a] TMA results from Table 2. NT, not tested.
